# Are we underestimating the diversity and incidence of insect bacterial symbionts? A case study in ladybird beetles

**DOI:** 10.1098/rsbl.2007.0373

**Published:** 2007-09-18

**Authors:** Lucy A Weinert, Matthew C Tinsley, Matilda Temperley, Francis M Jiggins

**Affiliations:** 1 Institute of Evolutionary Biology, University of EdinburghEdinburgh EH9 3JT, UK; 2 School of Biological and Environmental Sciences, University of StirlingStirling FK9 4LA, UK

**Keywords:** Coccinellidae, *Wolbachia*, *Spiroplasma*, *Rickettsia*, Flavobacteria, male-killing

## Abstract

Vertically transmitted bacterial symbionts are common in arthropods. However, estimates of their incidence and diversity are based on studies that test for a single bacterial genus and often only include small samples of each host species. Focussing on ladybird beetles, we collected large samples from 21 species and tested them for four different bacterial symbionts. Over half the species were infected, and there were often multiple symbionts in the same population. In most cases, more females than males were infected, suggesting that the symbionts may be sex ratio distorters. Many of these infections would have been missed in previous studies as they only infect a small proportion of the population. Furthermore, 11 out of the 17 symbionts discovered by us were either in the genus *Rickettsia* or *Spiroplasma*, which are rarely sampled. Our results suggest that the true incidence and diversity of bacterial symbionts in insects may be far greater than previously thought.

## 1. Introduction

Symbiotic bacteria that are transmitted vertically from mother to offspring are common among arthropods. Some of these associations are essential for host survival and can persist for millions of years (Chen *et al*. 1999). Some are facultative mutualists, such as symbionts that make their hosts resistant to parasitoids (Oliver *et al*. 2003). Others manipulate host reproduction to enhance their transmission, for example by distorting the host's sex ratio towards females, the sex that will transmit them to future generations. The discovery that symbionts in the genus *Wolbachia* infect approximately 17% of insect species (Werren *et al*. 1995) prompted more surveys of bacterial diversity in arthropods. These studies confirmed the original finding that *Wolbachia* is common, found that other symbionts such as *Cardinium* are widespread (Zchori-Fein & Perlman 2004), and revealed that many *Wolbachia* strains infect only a small proportion of the host population (Jiggins *et al*. 2001).

Current knowledge of symbiont diversity may be unreliable. Previous surveys have generally tested small samples of many species for just a single bacterial taxon (Werren *et al*. 1995; Zchori-Fein & Perlman 2004). This approach provides little information about low prevalence bacteria or the number of different symbionts harboured by each species. Other studies have surveyed only a few host species, preventing assessment of interspecific diversity (Haynes *et al*. 2003). Screens have also tested primarily for *Wolbachia* or *Cardinium*, whereas the diversity of symbiotic associations is probably far greater. Although it is more time consuming, testing large samples of each host species for a range of bacteria is necessary to accurately assess symbiont diversity both within single species and between them.

We investigated the diversity of bacterial symbionts in ladybird beetles (Coccinellidae). Ladybirds are particularly predisposed to male-killing bacteria as they lay their eggs in clutches and sibling cannibalism is common (Hurst & Jiggins 2000). Male-killers are bacteria that kill male hosts as embryos. As males rarely transmit vertically inherited symbionts, they provide no benefit to the bacteria. Male-killers can invade populations if females benefit when their brothers from the same brood are killed. Benefits include reduced sibling competition, inbreeding avoidance, evading cannibalism by brothers and opportunities to consume male eggs. Since these factors are determined by host ecology, male-killer distribution is thought to be driven by ecological parameters.

In previous studies, ladybird male-killers were identified by detecting skewed offspring sex ratios and then testing for the presence of bacteria (Hurst *et al*. 1996). This approach has revealed male-killers from four different bacterial groups (*Wolbachia*, *Rickettsia*, *Spiroplasma* and Flavobacteria species) in 10 species of ladybird (Majerus 2006). Current records report that *Wolbachia* infects one species; *Rickettsia* and *Spiroplasma* each infect three species; while five species harbour Flavobacteria. However, the diversity of male-killers in ladybirds remains unclear. Typically, only small samples have been screened as breeding is labour intensive, so low prevalence male-killers will remain undetected. Furthermore, there is a publication bias towards reporting positive results. In this study we used PCR to screen large samples of 21 different species for the four bacterial groups known to cause male-killing in ladybirds, giving us a unique picture of symbiotic diversity.

## 2. Material and methods

### (a) Ladybirds

Twenty-one ladybird species were collected from the locations in table 1 by beating vegetation while holding a collection tray underneath or sweeping vegetation with nets (thereby eliminating visual bias in collection rates). The ladybird species sampled solely reflects their ease of collection. All species samples contained 20 females or more, providing a 90% chance of detecting infections at 12% prevalence or over; in most cases considerably larger sample sizes were used.

Sex was determined using morphology of the posterior abdominal tergite or the presence/absence of a sclerotized sipho seen with an underlighted microscope; criteria were verified by genital dissection. Sterile blades were used to remove an abdominal section for DNA extraction and the remainder was preserved in ethanol.

### (b) DNA extraction and PCR

DNA was extracted as in Jiggins & Tinsley (2005) or using DNeasy columns for animal tissues (Qiagen, Valencia, CA). Samples extracted using columns were pooled with five ladybirds per column. The ribosomal DNA ITS region was amplified using BD1 and 4S (von der Schulenburg *et al*. 2001) to verify successful DNA extraction. Samples failing to yield a PCR product were discarded. Samples were then tested for *Wobachia* presence using *wsp*81f and *wsp*691r; *Rickettsia* using RSSUF and RSSUR; *Spiroplasma* using HaIn1 (specifically targets *Spiroplasma ixodetis* group) and MGSO; and Flavobacteria species using FL1 and FL2 (Tinsley & Majerus 2006). Positive bacterial controls were used in all PCRs. Pooled samples that tested positive for any bacteria were then extracted separately to measure bacterial prevalence. Since accurate DNA extraction for individual ladybirds in these samples cannot be confirmed, our bacterial prevalence estimates are conservative. At least one PCR product from each primer pair in each host species was sequenced to verify its identity (L. Weinert 2006, unpublished data).

## 3. Results

### (a) Symbiont diversity

We tested 2149 ladybirds from 21 different species for the presence of four bacterial genera that are known to cause male-killing in ladybirds (table 1). Over half the species (11 out of 21) were infected with at least one of the symbionts. *Rickettsia* was found in eight species, *Wolbachia* in six and *Spiroplasma* in three species. No species were infected with Flavobacteria. The relative frequency of ladybird species infected by each of the four bacteria was significantly different from what has previously been found (*Χ*_3_^2^=10.3; *p*=0.016; previous work described in §1). On average, each host species was infected by 0.8 different symbionts.

Of the 11 infected species, six were infected by two different symbionts and, in all cases, both bacteria were found in a single population. These double infections fell into two categories. In four of the ladybird species, the two different bacteria never infected the same individual. However, in *Rhyzobius (Rhizobius) litura* and *Coccidula rufa* both singly and doubly infected individuals were found.

### (b) Bacterial prevalence

The bacterial prevalence was very variable, ranging from 1 to 89%, with a median of 5%. There are striking differences in the prevalence of symbionts in males and females (table 1). Nine of the symbionts occurred only in females, compared with one that was only in males. Allowing a false discovery rate of 10% to correct for multiple tests (Benjamini & Hochberg 1995), out of the 19 different infected populations, five had a significantly higher bacterial prevalence in females, whereas one population had a higher prevalence in males (populations of the same species with significantly different prevalence treated separately).

*Rhyzobius (Rhizobius) litura* and *C. rufa* populations from Germany had much higher prevalence levels than other species, with nearly all individuals infected with *Rickettsia*, *Wolbachia* or both bacteria. These populations were also unusual in containing large numbers of infected males (although the prevalence is generally still highest in females).

### (c) Population sex ratio

Of the 28 different populations of ladybirds, eight were female biased and one was significantly male biased. If the sex ratio is determined by male-killers, then the number of uninfected females will be the same as the number of uninfected males. If there is a large excess of uninfected females, this suggests that there are other factors biasing the sex ratio, such as undiscovered male-killers. We tested whether the ratio of uninfected-males to uninfected-females differed from 1 : 1 (table 1). Six out of 28 populations still had a significant excess of uninfected females, and two had an excess of uninfected males (10% false discovery rate).

## 4. Discussion

*Rickettsia*, *Wolbachia* and *Spiroplasma* bacteria are all common among ladybirds. This is the first time that the incidence of *Spiroplasma, Rickettsia* or *Flavobacteria* in insects has been studied extensively, and our results suggest that some of these neglected groups of symbionts may be as common as *Wolbachia*. Many of the symbionts infect a small proportion of the population, and would have been missed by studies that examine only a few individuals of each species. It is therefore probable that both the taxonomic diversity of symbionts and the proportion of insect species infected by symbionts are far greater than previously suspected.

Symbiont diversity may actually be even greater than what our data suggest. In our largest samples, we detected bacteria that infect less than 1% of individuals; these would have been missed in our smaller samples. Furthermore, we did not test for the presence of all known symbiont taxa. The bacterial prevalence was also insufficient to explain the population sex ratio biases observed. This suggests that there may still be undiscovered sex ratio distorters from different bacterial taxa. However, male ladybirds suffer greater overwintering mortality than females, which may also cause this pattern.

Why have these symbionts spread through ladybird populations? In most cases, more females than males were infected, suggesting that they are sex ratio distorters. As all sex ratio distorters known in ladybirds are male-killers, it is possible that our symbionts are also male-killers. A surprising finding was that many of the bacteria also occur at a lower frequency in males. There have been few studies of whether males can survive male-killer infection in the wild. However, infected males survive at high temperatures in *Drosophila*, which is most likely caused by reduced bacterial density (Hurst *et al*. 2000). The widespread occurrence of infected males in our dataset could result from nuclear genes suppressing the male-killing phenotype, or from environmental effects.

Single populations commonly harboured more than one bacterial taxon. Theory predicts that this will be rare unless negatively frequency-dependent selection maintains the different bacteria (Randerson *et al*. 2000). There is evidence that natural selection maintains multiple male-killers in the ladybird *Adalia bipunctata* (Jiggins & Tinsley 2005). The finding that this pattern is common lends strength to the hypothesis that bacterial symbionts may commonly be maintained in populations either by negative frequency-dependent selection or because different strains are favoured in different populations.

The frequency with which the four symbionts occur across different species of ladybird was significantly different from that of previous studies of male-killers in ladybirds, which probably reflects the different screening methods. Interestingly, in previous work Flavobacteria were the commonest male-killers, while in our study they were absent. This could be a consequence of different sampling strategies, if Flavobacteria occur at a higher prevalence, or in different ladybird species or geographical areas than the other bacteria.

There is an unusual pattern of bacterial infection in *R. litura* and *C. rufa*. These species have female-biased population sex ratios, and their symbionts occur predominantly in females, suggesting that they are sex ratio distorters. They are, however, atypical of male-killers. Up to 60% of males were infected, suggesting that sex ratio distortion is inefficient. In addition, many individuals were co-infected with *Wolbachia* and *Rickettsia*: two different male-killers that have never been reported from a single individual before. One explanation is that there is partial suppression of the male-killing phenotype.

In conclusion, intensive sampling has uncovered widespread and extensive diversity of bacterial symbionts within one insect clade. Our findings demonstrate that the methods employed in previous studies may be biasing the picture of symbiont diversity. Efforts such as ours to uncover infection diversity both between and within species may provide more information about what determines symbiont distribution and how they spread through populations. We focused on a group of beetles that are known to be predisposed to male-killers, and further studies are needed to test if our results can be generalized across all insects.

## Figures and Tables

**Table 1:** Bacterial symbionts detected in ladybird beetle populations. (^*^p<0.05, ^**^p<0.01 and ^***^p<0.001 significance values are uncorrected for multiple tests.)

species name	location^a^	sample size	sex ratio^b^	sex ratio^b^ uninfected	bacteria	prevalence in females^c^	prevalence in males^c^
(*a*) *infected*							
*Adalia 2-punctata*^d^	Edinburgh, UK	84	0.27^***^	0.35^*^	*Spiroplasma*	0.28	0.04^*^
					*Rickettsia*	0.07	0.00
	Queenstown, New Zealand	70	0.50	0.56	*Spiroplasma*	0.29	0.09
*Anisosticta 19-punctata*^d^	Essex, UK	46	0.37	0.50	*Spiroplasma*	0.41	0.00^**^
	Ploen, Germany	123	0.46	0.46	uninfected		
*Adalia 10-punctata*^d^	Edinburgh, UK	112	0.52	0.52	*Rickettsia*	0.02	0.00
	Piedmonte, Italy	46	0.41	0.44	*Rickettsia*	0.11	0.00
*Coccinella 7-punctata*	Dunwich and Edinburgh, UK	115	0.47	0.49	*Wolbachia*	0.05	0.00
*Subcoccinella 24-punctata*	Braintree, UK	220	0.51	0.46	*Rickettsia*	0.04	0.15^*^
*Scymnus frontalis*	UK and Germany	35	n/a		*Rickettsia*	0.24	0.10
*Halyzia sedecimguttata*	Ploen, Germany	260	0.38^***^	0.39^***^	*Rickettsia*	0.01	0.00
					*Wolbachia*	0.02	0.00
	Somerset, UK	24	0.50	0.50	uninfected		
*Calvia quattuordecimguttata*	Ploen, Germany	57	0.49	0.49	*Rickettsia*	0.03	0.00
					*Wolbachia*	0.00	0.04
*Chilocorus bipustulatus*	Verona, Italy	20	0.40	0.41	*Wolbachia*	0.08	0.00
					*Spiroplasma*	0.08	0.13
	Hathersage, UK	15	0.40	0.40	uninfected		
*Rhyzobius* (*Rhizobius*) *litura*	Ploen, Germany	70	0.37^*^	1^**^	*Rickettsia*	0.84	0.62^*^
					*Wolbachia*	0.89	0.15^***^
	Thetford, UK	6	n/a		uninfected		
*Coccidula rufa*	Ploen, Germany	49	0.35^*^	0.80	*Rickettsia*	0.59	0.41
					*Wolbachia*	0.78	0.18^***^
(*b*) *uninfected*							
*Aphidecta obliterata*	Edinburgh, UK	44	0.30^**^				
*Exochomus quadripustulatus*	Thetford, UK	95	0.63^*^				
*Tytthaspis 16-punctata*	Thetford, UK	53	0.55				
*Propylea 14-punctata*	various, UK	52	0.54				
*Anatis ocellata*	Edinburgh and Thetford, UK	65	0.31^*^				
(from pupa)	Thetford, UK	111	0.45				
*Myzia oblongoguttata*	Edinburgh, UK	85	0.49				
*Coccinella hieroglyphica*	Balmoral, UK	83	0.55				
*Harmonia 4-punctata*	Thetford, UK	33	0.30^*^				
*Coccinella miranda* Wollaston	Tenerife, Spain	146	0.53				
*Myrha octodecimguttata*	Edinburgh, UK	30	0.37				
	Murcia, Spain	67	0.27^***^				

a Populations where symbiont type, sex ratio or infection level did not significantly differ were pooled together.

b Proportion of males, deviations from a 1 : 1 sex ratio were tested using an exact binomial goodness of fit test.

c Difference in prevalence between males and females was tested using a exact test.

d Species where symbionts are already known to occur.
